# DUSP5-mediated inhibition of smooth muscle cell proliferation suppresses pulmonary hypertension and right ventricular hypertrophy

**DOI:** 10.1152/ajpheart.00115.2021

**Published:** 2021-06-18

**Authors:** Bradley S. Ferguson, Sara A. Wennersten, Kimberly M. Demos-Davies, Marcello Rubino, Emma L. Robinson, Maria A. Cavasin, Matthew S. Stratton, Andrew M. Kidger, Tianjing Hu, Stephen M. Keyse, Robert A. McKnight, Robert H. Lane, Eva S. Nozik, Mary C. M. Weiser-Evans, Timothy A. McKinsey

**Affiliations:** ^1^Division of Cardiology, Department of Medicine, University of Colorado Anschutz Medical Campus, Aurora, Colorado; ^2^Consortium for Fibrosis Research & Translation, University of Colorado Anschutz Medical Campus, Aurora, Colorado; ^3^Stress Response Laboratory, Division of Cellular Medicine, Jacqui Wood Cancer Centre, School of Medicine, University of Dundee, Dundee, United Kingdom; ^4^Department of Pediatrics, University of Utah, Salt Lake City, Utah; ^5^Children’s Mercy Kansas City, Kansas City, Missouri; ^6^Department of Pediatrics, University of Colorado Anschutz Medical Campus, Aurora, Colorado; ^7^Cardiovascular Pulmonary Research, University of Colorado Anschutz Medical Campus, Aurora, Colorado; ^8^Division of Renal Diseases and Hypertension, Department of Medicine, University of Colorado Anschutz Medical Campus, Aurora, Colorado

**Keywords:** angiotensin, ERK, kinase, pulmonary hypertension, smooth muscle cell

## Abstract

Pulmonary hypertension (PH) is associated with structural remodeling of pulmonary arteries (PAs) because of excessive proliferation of fibroblasts, endothelial cells, and smooth muscle cells (SMCs). The peptide hormone angiotensin II (ANG II) contributes to pulmonary vascular remodeling, in part, through its ability to trigger extracellular signal-regulated kinase (ERK1/2) activation. Here, we demonstrate that the ERK1/2 phosphatase, dual-specificity phosphatase 5 (DUSP5), functions as a negative regulator of ANG II-mediated SMC proliferation and PH. In contrast to wild-type controls, Dusp5 null mice infused with ANG II developed PH and right ventricular (RV) hypertrophy. PH in Dusp5 null mice was associated with thickening of the medial layer of small PAs, suggesting an in vivo role for DUSP5 as a negative regulator of ANG II-dependent SMC proliferation. Consistent with this, overexpression of DUSP5 blocked ANG II-mediated proliferation of cultured human pulmonary artery SMCs (hPASMCs) derived from patients with idiopathic PH or from failed donor controls. Collectively, the data support a role for DUSP5 as a feedback inhibitor of ANG II-mediated ERK signaling and PASMC proliferation and suggest that disruption of this circuit leads to adverse cardiopulmonary remodeling.

**NEW & NOTEWORTHY** Dual-specificity phosphatases (DUSPs) serve critical roles in the regulation of mitogen-activated protein kinases, but their functions in the cardiovascular system remain poorly defined. Here, we provide evidence that DUSP5, which resides in the nucleus and specifically dephosphorylates extracellular signal-regulated kinase (ERK1/2), blocks pulmonary vascular smooth muscle cell proliferation. In response to angiotensin II infusion, mice lacking DUSP5 develop pulmonary hypertension and right ventricular cardiac hypertrophy. These findings illustrate DUSP5-mediated suppression of ERK signaling in the lungs as a protective mechanism.

## INTRODUCTION

Pulmonary arterial hypertension (PAH), which is defined in humans as a resting mean pulmonary artery pressure of 20 mmHg or above, with pulmonary vascular resistance of three woods units, is a deadly disease (1). Broadly, pulmonary hypertension (PH) restricts blood flow through the pulmonary arterial circulation, leading to elevated pulmonary vascular resistance and often resulting in right ventricular (RV) heart failure. Pathogenic mechanisms in PH include aberrant vasoconstriction of pulmonary vasculature, as well as complex remodeling of intimal, medial, and adventitial layers of vessels within the lung (2). Current standard-of-care treatment for patients with PH is based on the use of vasoactive drugs, including endothelin receptor antagonists, phosphodiesterase-5 inhibitors, prostacyclins, and soluble guanylyl cyclase activators (3). However, given the pulmonary vascular remodeling component of PH, it is believed that more effective therapeutic strategies will be based on the combined use of vasodilators and drugs that target vascular cell proliferation and growth (2). Unfortunately, the one clinical study designed to specifically target vascular remodeling in PH using the tyrosine kinase inhibitor, imatinib, failed to meet the primary end point (4). Thus, the molecular mechanisms governing pulmonary vascular remodeling need to be better defined to guide development of novel, antiproliferative therapies for PH.

Excessive pulmonary vascular smooth muscle cell (VSMC) proliferation and hypertrophy have long been recognized as pathogenic mechanisms in PH (5). Thickening of the medial layer of muscular pulmonary arteries (PAs) and formation of occlusive lesions in normally nonmuscular small vessels increases pulmonary vascular resistance (2). A prominent intracellular signaling effector that lies downstream of pro-proliferative receptors in SMCs is extracellular signal-regulated kinase (ERK), which is a member of the mitogen-activated protein kinase (MAPK) family. Upon phosphorylation by MEK kinases, ERK1 and ERK2 family members stimulate cellular proliferation by phosphorylating a multitude of substrates, including the ETS-like 1 (ELK-1) transcription factor (6).

As a component of the renin-angiotensin-aldosterone system (RAAS), in addition to effects on blood volume and the autonomic nervous system, the peptide hormone angiotensin II (ANG II) contributes to the pathogenesis of cardiovascular diseases by stimulating VSMC proliferation, migration, contraction, and hypertrophy. The mitogenic action of ANG II on VSMCs is governed by signaling via the G protein-coupled receptor AT1, which triggers a series of intracellular signaling events, including ERK1/2 activation. Demonstration of efficacy of AT1 antagonists in preclinical models suggested a role for ANG II signaling in PH pathogenesis (7–10), and these findings have been corroborated by studies of human samples showing evidence of enhanced ANG II signaling in lungs of patients with PH (7, 11). However, the details of ANG II signaling in pulmonary vasculature remain poorly understood.

MAPKs are inactivated upon dephosphorylation by dual specificity MAPK phosphatases (DUSPs or MKPs) (12). Here, we demonstrate that DUSP5, an inducible nuclear MKP, which is highly specific for the classical ERK1/2 MAPKs (13), functions as a negative regulator of ANG II-mediated pulmonary artery SMC (PASMC) proliferation. DUSP5-deficient mice chronically infused with ANG II develop exaggerated medial thickening of PAs, with subsequent PH and RV hypertrophy, whereas wild-type control mice exhibit no changes in pulmonary hemodynamics in response to this growth factor. Together with findings employing cultured cells, these data suggest that DUSP5 functions as a negative regulator of PASMC proliferation, and that disruption of this circuit leads to adverse pulmonary vascular remodeling and PH.

## METHODS

### Animals

Animal care followed the Institute of Laboratory Animal Research Guide for the Care and Use of Laboratory Animals and was approved by the Institutional Animal Use and Care Committee at the University of Colorado Denver. Animal experiments were in accordance with NIH guidelines. Homologous recombination in mouse embryonic stem cells was performed using a targeting construct containing the *Dusp5* gene from the start of exon 2 to the stop codon in exon 4, which was modified to have a *loxP* site inserted upstream of exon 3 and a *PGK-Neo-loxP* cassette in intron 3. Knockout (KO) mice were generated by crossing to transgenic mice harboring a *CMV-Cre* transgene and were backcrossed to 129S6/SvEvTac mice (Taconic) for eight generations. Ten-week-old, male, wild-type (WT) and KO mice were used for all experiments. WT and KO animals were randomized to receive sham pumps or were infused with ANG II (1.0 µg/kg/min; Bachem) for 2 wk using osmotic minipumps (Alzet).

### Hemodynamic Analyses

Echocardiographic analyses were performed using a Vevo2100 instrument (VisualSonics). To obtain right ventricular outflow tract Pulsed-wave (PW) Doppler, a parasternal long axis of the left ventricle was obtained to view the pulmonary artery. PW Doppler mode was used to obtain waveforms at the level of the aortic valve, obtaining the highest velocity by color Doppler. Notch presence correlates with decreased pulmonary artery acceleration time (PAAT) and PAAT/PET (pulmonary artery acceleration time/pulmonary ejection time). Right ventricular systolic pressure (RVSP) was measured on the final day of the study using a solid-state catheter (Millar-ADI system); mice were ventilated with compressed air and 2% isoflurane (Hallowell). Systemic blood pressure was assessed in conscious animals using a noninvasive tail-cuff system (Coda, Kent Scientific). All mice were trained for 3 days before final measurements. Ten total blood pressure measurements were obtained and averaged for each mouse.

### Immunohistochemistry

Lungs were flushed with a cold PBS-heparin solution, and the left lobe was fixed in 4% paraformaldehyde and placed in a 30% sucrose solution overnight before being snap-frozen in a block containing optimum cutting temperature compound. Vessel wall thickness was assessed as previously described (14). A minimum of four mice per group and 20 arterioles (<100 µm) per mouse were quantified and averaged.

### Cell Culture

Human PASMCs were provided as de-identified samples from a patient with idiopathic pulmonary arterial hypertension (IPAH), or a failed donor (FD) control, by the PHBI Research Network and maintained in Sm-GM2 medium (Lonza) at 37°C in a 5% CO_2_ humidified incubator. Some experiments employed human pulmonary artery smooth muscle cells (hPASMCs) from ScienCell (No. 3110), cultured in medium from the same company (No. 1101). Human PASMCs were used at less than passage 9 for all studies. Prior to treatment with ANG II (1 µM; Bachem), hPASMCs were cultured in medium containing 0.1% FBS for 18 h to induce growth arrest. Cells were pretreated with 5 µM PD98059 (LC Labs; P-4313) or 10 µM U0126 (Fisher; PR-V1121) for 30 min before ANG II addition. Studies using human PASMCs were deemed Institutional Review Board exempt.

### Immunoblotting and Indirect Immunofluorescence

Membranes were probed with primary antibodies for phosphorylated ERK (Cell Signaling Technology; 4370), total ERK (Santa Cruz Biotechnology; sc-153 or Cell Signaling Technology; 4696), sheep polyclonal anti-DUSP5 (13), rabbit anti-DUSP5 (Abcam; ab200708), c-Myc (Santa Cruz Biotechnology; sc-40), β-tubulin (Sigma; T8328), or α-tubulin (Santa Cruz Biotechnology; sc-23948 HRP). Horseradish peroxidase (HRP)-conjugated secondary antibodies (Southern Biotech) were used at a concentration of 1:2,000. SuperSignal West Pico chemiluminescence system (Thermo Scientific) and a FluorChem HD2 Imager (Alpha Innotech) were used to detect proteins. For indirect immunofluorescence, anti-DUSP5 antibody (Abcam) was diluted 1:100 in phosphate-buffered saline with 0.1% Tween-20 (PBS-T) containing 1% BSA and incubated overnight. Secondary goat anti-rabbit IgG conjugated to Alexa Fluor 594 (Thermo Fisher; A-11012) and DAPI were used for detection of DUSP5 and nuclei, respectively.

### Real-Time qRT-PCR

Total RNA was isolated and analyzed to quantify mouse *Dusp5* (Fwd: TCGCCTACAGACCAGCCTAT and Rev: GTAGTGTAGGTGGGTGGTGC) and human *Dusp5* (Fwd: 5′- TCAGCCAGTGTGGAAAACCAG-3′, Rev: 3′- AGGCACTTCCAAGGTAGAGGA-5′) mRNA levels. Data were expressed as 2^−ΔΔCt^ and normalized to vehicle control. Data were normalized to 18S (Fwd: GCCGCTAGAGGTGAAATTCTTG and Rev: CTTTCGCTCTGGTCCGTCTT).

### Statistical Analyses

Statistical analyses were completed by ANOVA followed by post hoc testing (Tukey’s test) using GraphPad Prism software. Statistical significance (defined as *P* < 0.05) is reported.

## RESULTS AND DISCUSSION

**Figure 1.**
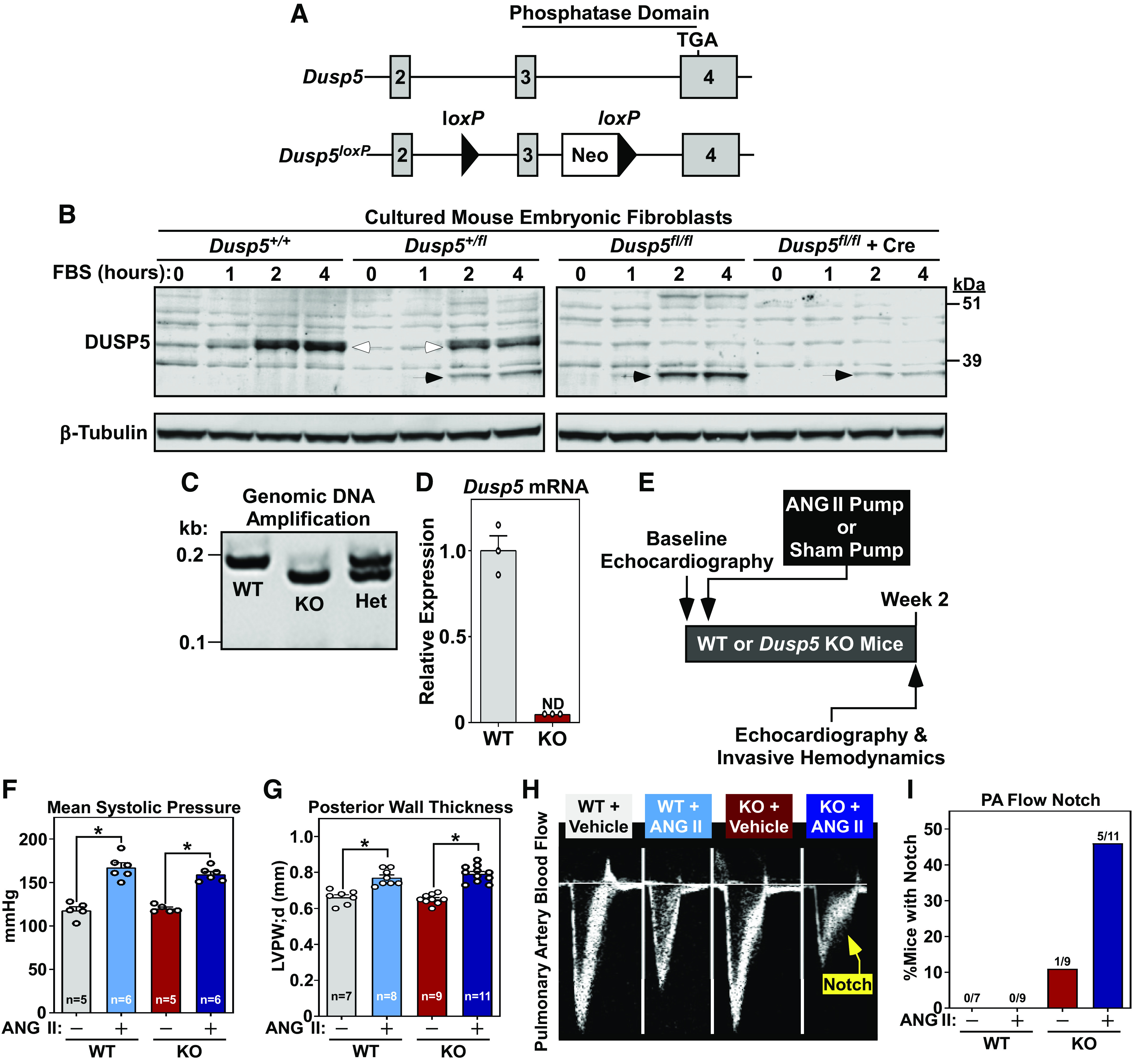
DUSP5-deficient mice exhibit signs of reduced pulmonary vascular compliance. *A*: schematic representation of the wild-type (WT) and targeted knockout (KO) *Dusp5* gene locus. *B*: mouse embryonic fibroblasts (MEFs) were isolated from WT mice (*Dusp5^+/+^*), heterozygous floxed *Dusp5* mice (*Dusp5^+/fl^*), or homozygote floxed *Dusp5* mice (*Dusp5^fl/fl^*). Cells were cultured, subjected to serum deprivation, then stimulated with FBS for the indicated times; some *Dusp5*^fl/fl^ MEFs were infected with adenovirus-encoding Cre. Cell lysates were subjected to immunoblotting to detect endogenous DUSP5 and β-tubulin. Full-length and truncated DUSP5 are indicated with open and closed arrows, respectively. *C*: PCR amplification of genomic DNA to assess floxed, WT, or KO alleles. *D*: quantitative PCR to detect *Dusp5* mRNA expression in hearts of WT and KO animals. *E*: experimental design. *F*: tail-cuff measurements of mean systolic pressure following 2 wk of ANG II infusion and in sham controls. *G*: echocardiographic assessment of left ventricular posterior wall (LVPW) thickness. For *F* and *G*, *P < 0.05 vs. sham controls. *H*: Pulse-wave Doppler assessment of pulmonary outflow. In KO mice treated with ANG II, there was evidence of transient cessation of forward pulmonary blood flow during systole, which was revealed as a “notch” in the signal. *I*: percentage of mice in each group with a detectable notch in pulmonary blood flow. DUSP5, dual-specificity phosphatase 5; ND, not detected; PA, pulmonary artery.

DUSP5 has previously been shown to reduce VSMC contractile capability and blunt myogenic tone of arteries (15, 16). To address whether DUSP5 regulates SMC function in vivo, mice harboring *loxP* sites flanking exon 3 of the *Dusp5* gene were generated (Fig. 1*A*). For unknown reasons, the presence of the *loxP* sites led to aberrant expression of a lower molecular weight form of DUSP5 protein (Fig. 1*B*). As such, studies were limited to mice with global deletion of *Dusp5* (Fig. 1, *C* and *D*). *Dusp5* KO mice and littermate controls were implanted with subcutaneous osmotic minipumps to release ANG II for 2 wk; control animals received sham pumps (Fig. 1*E*). WT and *Dusp5* KO mice developed systemic hypertension equivalently (Fig. 1*F*), and associated left ventricular hypertrophy was comparable in both sets of mice (Fig. 1*G*). However, upon Doppler evaluation of pulmonary artery flow, we noted a “notch” in the signal in KO mice treated with ANG II (Fig. 1*H*), which is indicative of a transient cessation of forward PA blood flow during systole due to reduced pulmonary vascular compliance. Notching was observed in >40% of KO mice subjected to ANG II, but was not seen in WT mice in the absence or presence of ANG II; only one KO mouse exhibited notching without ANG II (Fig. 1I).

**Figure 2.**
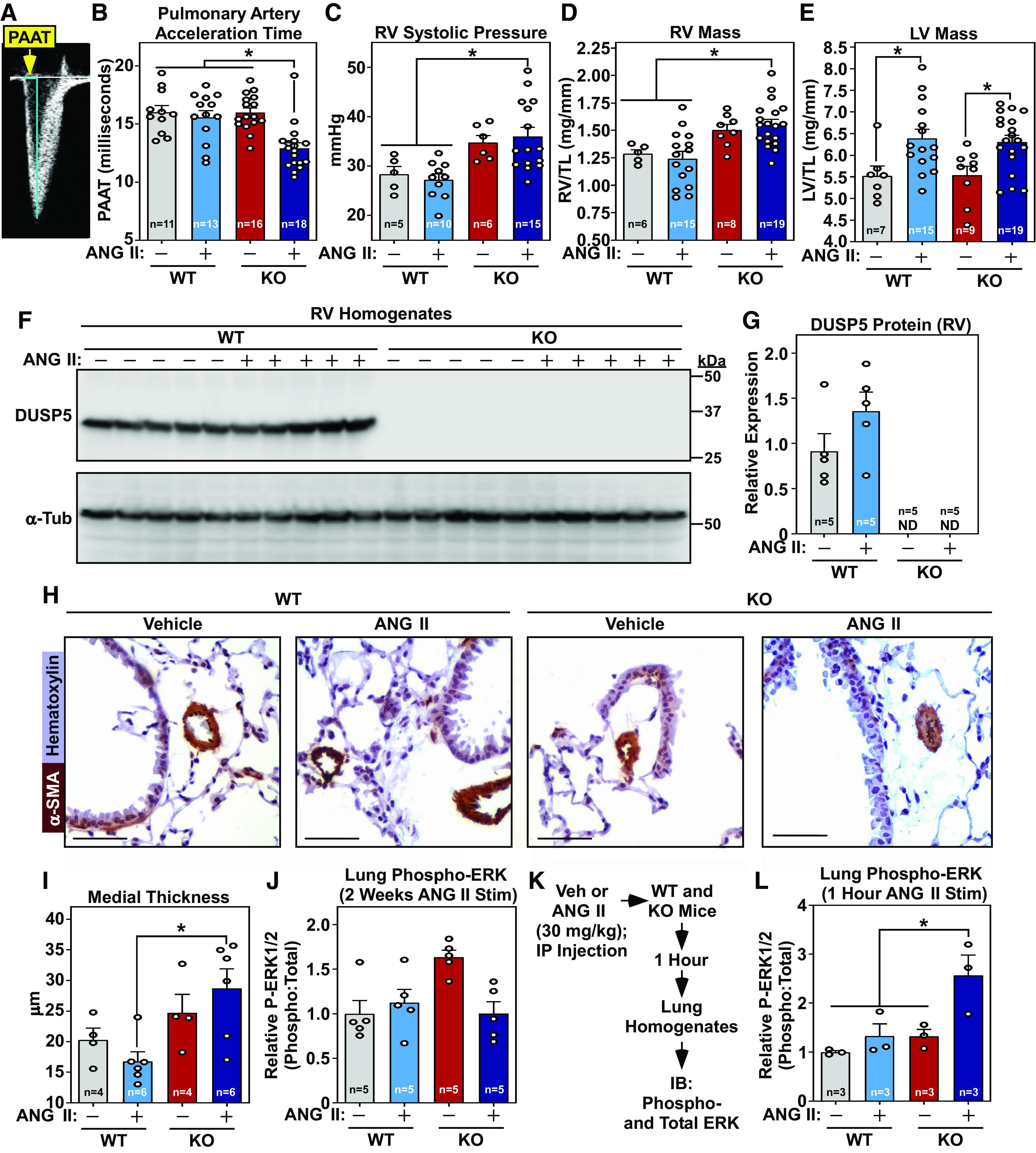
DUSP5-deficient mice treated with ANG II develop PH in association with pulmonary vascular remodeling. Wild-type (WT) and *Dusp5* knockout (KO) mice were infused with ANG II for 2 wk. *A*: a Doppler image of pulmonary outflow indicates the region that was used to calculate pulmonary artery acceleration time (PAAT). *B*: KO mice treated with ANG II had significantly reduced PAAT, indicating elevated PA pressure. Each point represents data from an individual mouse. *C*: RVSP was quantified at study end point using an invasive catheter. *D* and *E*: RV and LV hypertrophy was assessed by quantified ventricular mass-to-tibia length ratios. *F*: RV homogenates were subjected to immunoblotting with anti-DUSP5 and anti-α-tubulin antibodies. *G*: densitometry quantification of RV DUSP5 protein expression normalized to α-tubulin levels. *H*: representative images of sectioned lungs stained for α-smooth muscle actin (α-SMA; brown) and hematoxylin (purple); scale bar = 50 µm. *I*: quantification of PA medial thickness. *J*: quantification of immunoblots to assess ERK1/2 phosphorylation, relative to total ERK1/2, in lungs of mice from the 2-wk ANG II infusion study. *K*: schematic depiction of the acute ANG II experiment. *L*: quantification of immunoblots to assess ERK1/2 phosphorylation, relative to total ERK1/2, in lungs of mice from the 1-h ANG II study. *P < 0.05 vs. the indicated groups; *n*, number of animals analyzed per group. DUSP5, dual-specificity phosphatase 5; ERK1/2, extracellular signal-regulated kinase; LV, left ventricular; ND, not detected; PA, pulmonary artery; PH, pulmonary hypertension; RV, right ventricular; RVSP, right ventricular systolic pressure.

A follow-up study was performed to further evaluate the possibility that deletion of DUSP5 leads to altered pulmonary hemodynamics, initially employing Doppler imaging to noninvasively quantify pulmonary artery acceleration time (PAAT) as the primary end point (Fig. 2*A*). PAAT was significantly reduced in KO mice subjected to ANG II (Fig. 2*B*), suggesting that these mice have increased pulmonary arterial pressure. In contrast, PAAT was equivalent in WT mice in the absence or presence of ANG II, and in KO mice without ANG II (Fig. 2*B*). KO mice treated with ANG II had elevated right ventricular systolic pressure (RVSP) (Fig. 2*C*) and RV hypertrophy (Fig. 2*D*), both of which are indicative of PH. In accordance with the echocardiographic data (Fig. 1*G*), morphometric analysis of left ventricles (LVs) demonstrated ANG II-mediated hypertrophy that was equal between WT and KO mice (Fig. 2*E*), illustrating that DUSP5 deletion selectively augments RV hypertrophy in response to ANG II stimulation. Immunoblotting confirmed the absence of DUSP5 protein in RVs of KO mice (Fig. 2, *F* and *G*). The failure of DUSP5 deletion to exacerbate LV hypertrophy is surprising given our prior demonstration that this phosphatase suppresses cardiomyocyte growth (17) and suggests that other DUSPs compensate for the lack of DUSP5 in the LV in vivo. Histological analysis revealed thickening of the medial layer of small PAs of KO mice treated with ANG II (Fig. 2, *H* and *I*), suggesting that adverse pulmonary vascular remodeling contributes to the development of PH in these mice.

Immunoblotting failed to reveal altered ERK1/2 phosphorylation in response to 2 wk of ANG II administration in lungs of WT or KO mice (Fig. 2*J*). To determine whether DUSP5 deletion transiently exacerbates ANG II-mediated ERK1/2 phosphorylation in the lung, an acute study was performed with WT and KO mice administered ANG II or vehicle control by intraperitoneal injection (Fig. 2*K*). After 1 h of treatment, lungs were harvested for ERK1/2 phosphorylation analysis. As shown in Fig. 2*L*, acute ANG II treatment stimulated ERK1/2 phosphorylation in KO but not WT mouse lungs. These findings suggest that DUSP5 deletion promotes ERK signaling in the lung early after ANG II treatment, which could explain the PA medial thickening and PH observed in KO mice.

**Figure 3.**
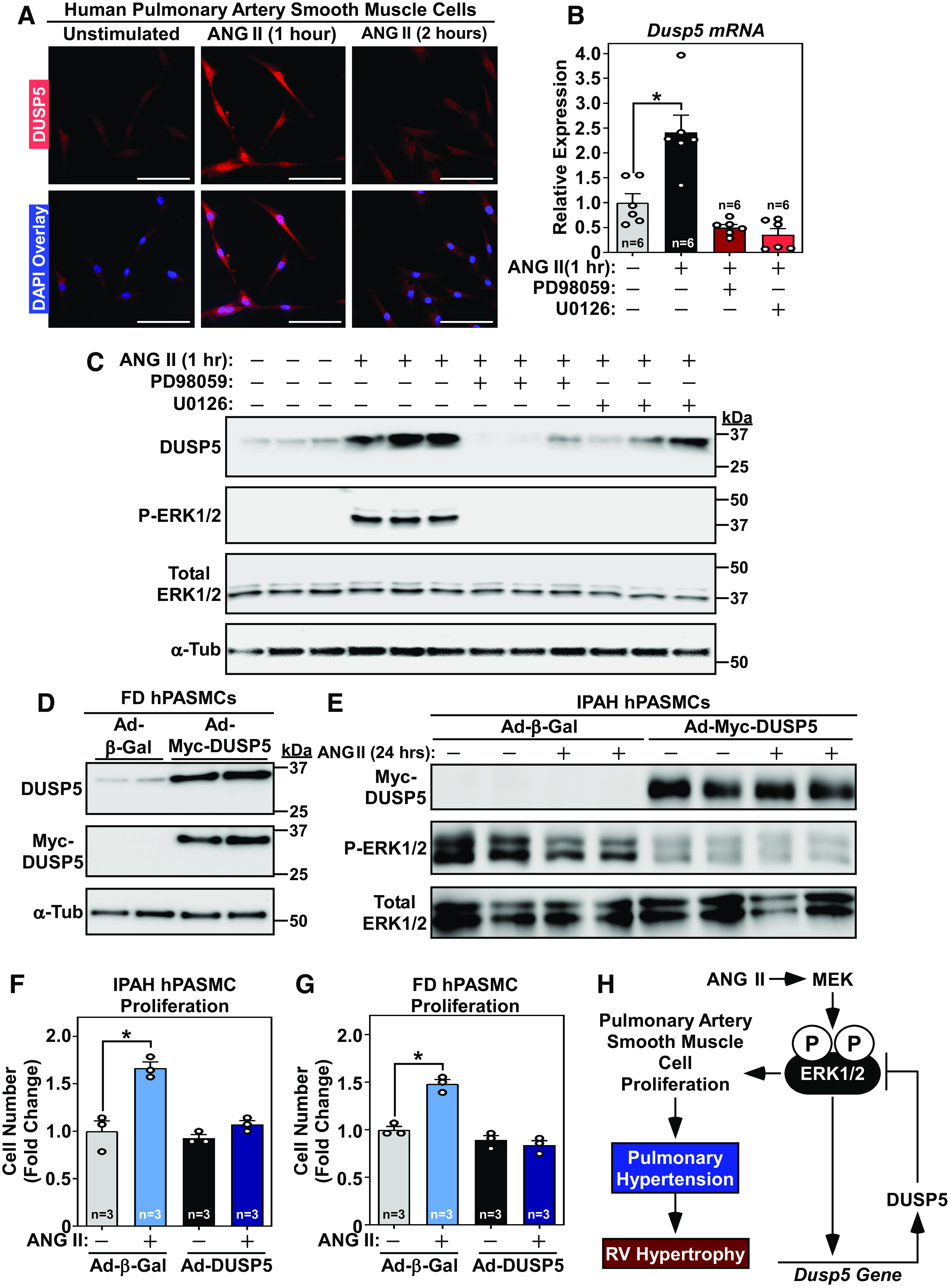
DUSP5 inhibits ERK1/2 phosphorylation and proliferation in human pulmonary artery smooth muscle cells. A: indirect immunofluorescence staining of DUSP5 in cultured normal human pulmonary artery smooth muscle cells (hPASMCs) stimulated with ANG II for the indicated times. DAPI overlay reveals prominent nuclear DUSP5 in cells stimulated with ANG II for 1 h; scale bar = 100 µm. *B*: quantitative PCR analysis of *Dusp5* mRNA expression in normal hPASMCs treated with ANG II for 1 h in the absence or presence of the indicated MEK inhibitors. *P < 0.05 vs. unstimulated; *n* = 3 plates of cells from two different experiments combined. *C*: immunoblotting using homogenates from normal hPASMCs treated as indicted. *D*: normal hPASMCs from a failed donor (FD) were infected with Ad-β-galactosidase or Ad-Myc-DUSP5 for 24 h prior to harvesting for immunoblotting with the indicated antibodies. *E*: PASMCs from an individual with idiopathic pulmonary arterial hypertension (IPAH) were infected with Ad-β-galactosidase or Ad-Myc-DUSP5 for 24 h prior to harvesting for immunoblotting to detect Myc-DUSP5, P-ERK1/2, and total ERK1/2. *F*: cells were counted from parallel plates of cells following 24 h of treatment. *G*: FD PASMCs were infected and analyzed as in *F*. For *F* and *G*, *P < 0.05 vs. unstimulated Ad-β-galactosidase-infected cells; *n* = 3 plates of cells per condition. *H*: a model depicting the regulation and function of DUSP5 in PASMCs. DUSP5, dual-specificity phosphatase 5; ERK1/2, extracellular signal-regulated kinase; PASMCs, pulmonary artery smooth muscle cells.

Experiments were performed to more specifically address DUSP5 regulation and function in PASMCs, as well as the translational relevance of our findings. Treatment of cultured normal hPASMCs with ANG II for 1 h led to enrichment of DUSP5 in nuclei of the cells, which dissipated at 2 h posttreatment (Fig. 3*A*). Quantitative PCR analysis of RNA from independent cultures of cells demonstrated ANG II-mediated induction of *Dusp5* mRNA expression, which was blocked by small molecule inhibitors of the upstream ERK-activating kinases, MEK1 and MEK2, reflecting a negative feedback loop for inhibition of ERK signaling (Fig. 3*B*). Similarly, immunoblotting demonstrated ANG II-induced, MEK-dependent, expression of DUSP5 protein in hPASMCs (Fig. 3*C*). To address the relevance of these findings to human disease, PASMCs from a patient with IPAH, or a FD control, were cultured and infected with adenoviruses encoding DUSP5 or β-galactosidase as a negative control (multiplicity of infection = 10) (17). Densitometry analysis of an immunoblot with FD cell lysates demonstrated that ectopic DUSP5 was expressed approximately sixfold higher than endogenous DUSP5 (Fig. 3*D* and data not shown). In IPAH PASMCs, ectopic DUSP5 reduced ERK1/2 phosphorylation (Fig. 3*E*); of note, after 24 h of treatment, ANG II-mediated ERK1/2 phosphorylation was not evident, further suggesting transient activation of the pathway by the growth factor. Additionally, DUSP5 overexpression blocked ANG II-induced proliferation of IPAH hPASMCs and FD hPASMCs (Fig. 3, *F* and *G*).

Collectively, the findings presented here reveal an unexpected role for DUSP5 as a negative regulator of PASMC proliferation and lung vascular remodeling in response to ANG II signaling. Whether the results are related to the recent demonstration of *Dusp5* gene single-nucleotide polymorphisms in patients with PH and bronchopulmonary dysplasia remains to be determined (18). Our data support a model in which DUSP5 functions as a feedback inhibitor of ERK1/2 signaling in PASMCs, with elimination of DUSP5 leading to disruption of this circuit and resulting in aberrant PASMC proliferation, PH and RV hypertrophy (Fig. 3*H*). It appears that lung vasculature is more sensitive to DUSP5 deletion than systemic vasculature, since KO mice developed PH but not exaggerated systemic hypertension in response to ANG II. Given that the lung is the major site of ANG II production due to high-level expression of angiotensin-converting enzyme (19), we posit that DUSP5 serves a particularly important role in the pulmonary vascular bed to prevent spurious PASMC proliferation in the face of the locally elevated concentration of ANG II in the pulmonary circulation.

## DATA AVAILABILITY

Data are available on request from the authors.

## GRANTS

Work in the Keyse laboratory was supported by Cancer Research UK Programme Grant C8227/A12053, MRC Research Grant MR/N020790/1, and a Dundee Cancer Centre Studentship (to A.M.K.). T. A. McKinsey was supported by the National Institutes of Health (NIH) Grants HL116848, HL147558, DK119594, HL127240, and HL150225 and American Heart Association (AHA) Grant 16SFRN31400013. M. Rubino and E. L. Robinson were supported by the AHA Grants 20POST35210627 and 829504, respectively. Echocardiography was supported by NIH Grant 1S10OD018156-01, entitled “Small Animal Ultrasound Imager-Vevo 2100.” PHBI cells were funded by NIH Grant RO3 HL110783 (to E.S.N.).

## DISCLOSURES

T. A. McKinsey is on the S.A.B. of Artemes Bio received funding from Italfarmaco for an unrelated project and has a subcontract from Eikonizo Therapeutics for a Small Business Innovation Research grant from the National Institutes of Health (HL154959). None of the other authors has any conflicts of interest, financial or otherwise, to disclose.

## AUTHOR CONTRIBUTIONS

B.S.F. and T.A.M. conceived and designed research; B.S.F., S.A.W., K.M.D-D., M.R., E.L.R., M.S.S., A.M.K., T.H., and R.A.M. performed experiments; B.S.F., S.A.W., K.M.D-D., E.L.R., M.A.C., M.S.S., A.M.K., S.M.K., and T.A.M. analyzed data; B.S.F., S.A.W., M.A.C., S.M.K., M.C.M.W-E., and T.A.M. interpreted results of experiments; B.S.F., R.A.M., and T.A.M. prepared figures; B.S.F., R.A.M., and T.A.M. drafted manuscript; E.S.N. and M.C.M.W-E. edited and revised manuscript; B.S.F., S.A.W., K.M.D-D., M.R., E.L.R., M.A.C., M.S.S., T.H., S.M.K., R.A.M., R.H.L., E.S.N., M.C.M.W-E., and T.A.M. approved final version of manuscript.
